# Frontiers and hotspots evolution in anti-inflammatory studies for coronary heart disease: A bibliometric analysis of 1990–2022

**DOI:** 10.3389/fcvm.2023.1038738

**Published:** 2023-02-16

**Authors:** Jiale Zhang, Chenyang Ji, Xu Zhai, Hongxuan Tong, Jingqing Hu

**Affiliations:** ^1^Institute of Basic Theory for Chinese Medicine, China Academy of Chinese Medical Sciences, Beijing, China; ^2^Science and Technology College of Jiangxi University of Traditional Chinese Medicine, Nanchang, Jiangxi, China; ^3^Graduate School of China Academy of Chinese Medical Sciences, Beijing, China

**Keywords:** anti-inflammatory, coronary heart disease, atherosclerosis, C-reactive protein, bibliometric analysis, inflammation

## Abstract

**Background:**

Coronary heart disease (CHD) is characterized by forming of arterial plaques composed mainly of lipids, calcium, and inflammatory cells. These plaques narrow the lumen of the coronary artery, leading to episodic or persistent angina. Atherosclerosis is not just a lipid deposition disease but an inflammatory process with a high-specificity cellular and molecular response. Anti-inflammatory treatment for CHD is a promising therapy; several recent clinical studies (CANTOS, COCOLT, and LoDoCo2) provide therapeutic directions. However, bibliometric analysis data on anti-inflammatory conditions in CHD are lacking. This study aims to provide a comprehensive visual perspective on the anti-inflammatory research in CHD and will contribute to further research.

**Materials and methods:**

All the data were collected from the Web of Science Core Collection (WoSCC) database. We used the Web of Science’s systematic tool to analyze the year of countries/regions, organizations, publications, authors, and citations. CiteSpace and VOSviewer were used to construct visual bibliometric networks to reveal the current status and emerging hotspot trends for anti-inflammatory intervention in CHD.

**Results:**

5,818 papers published from 1990 to 2022 were included. The number of publications has been on the rise since 2003. Libby Peter is the most prolific author in the field. “Circulation” was ranked first in the number of journals. The United States has contributed the most to the number of publications. The Harvard University System is the most published organization. The top 5 clusters of keywords co-occurrence are inflammation, C-reactive protein, coronary heart disease, nonsteroidal anti-inflammatory, and myocardial infarction. The top 5 literature citation topics are chronic inflammatory diseases, cardiovascular risk; systematic review, statin therapy; high-density lipoprotein. In the past 2 years, the strongest keyword reference burst is “Nlrp3 inflammasome,” and the strongest citation burst is “Ridker PM, 2017 (95.12).”

**Conclusion:**

This study analyzes the research hotspots, frontiers, and development trends of anti-inflammatory applications in CHD, which is of great significance for future studies.

## Introduction

In recent decades, inflammation has been a growing concern in atherosclerotic coronary artery disease. Meanwhile, the basic and clinical anti-inflammation studies in coronary artery disease have been widely explored (1). In 1986, Professor Russell Ross (2) explicitly stated that atherosclerosis is an inflammatory disease and excessive defensive response to injury. In 1996, Michael A Mendall (3) investigated the relationship between chronic low-grade systemic inflammation (c-reactive protein, CRP) and coronary artery disease through a cross-sectional study. The results suggest that the body’s response to inflammation may affect the development of atherosclerosis in the middle-aged population. In 1999, John Danesh (4) explored the relevance of low-grade inflammatory processes to cardiovascular disease and vascular risk factors. It was concluded that hypersensitive C-reactive protein (hs-CRP) is a strong predictor of future cardiovascular events. CANTOS confirms the relationship between inflammation and coronary heart disease, and reducing inflammation reduces the risks of heart disease (5). This hypothesis has been continuously proven scientifically, from pathological studies of the blood vessel wall to epidemiological studies of circulating inflammatory factors in preliminary intervention studies. More studies are focusing on the mechanisms of anti-inflammatory action in coronary heart disease and exploring new therapeutic approaches for anti-inflammatory drugs (6–9).

Bibliometry is the cross-science of quantitatively analyzing all knowledge carriers using mathematical and statistical methods (10). Bibliometric analysis can capture literature groups’ characteristics and hot trends within a topic domain (11). Therefore, a comprehensive understanding can be gained using bibliometric analysis methods, which greatly help scientific research. In recent years, bibliometric analysis has played a role in medicine with the surge of medical papers (12). However, the bibliometric analysis of inflammation in CHD is still lacking. In this paper, we conducted bibliometric research on anti-inflammation use in CHD to explore its development trends.

## Materials and methods

**Figure 1:**
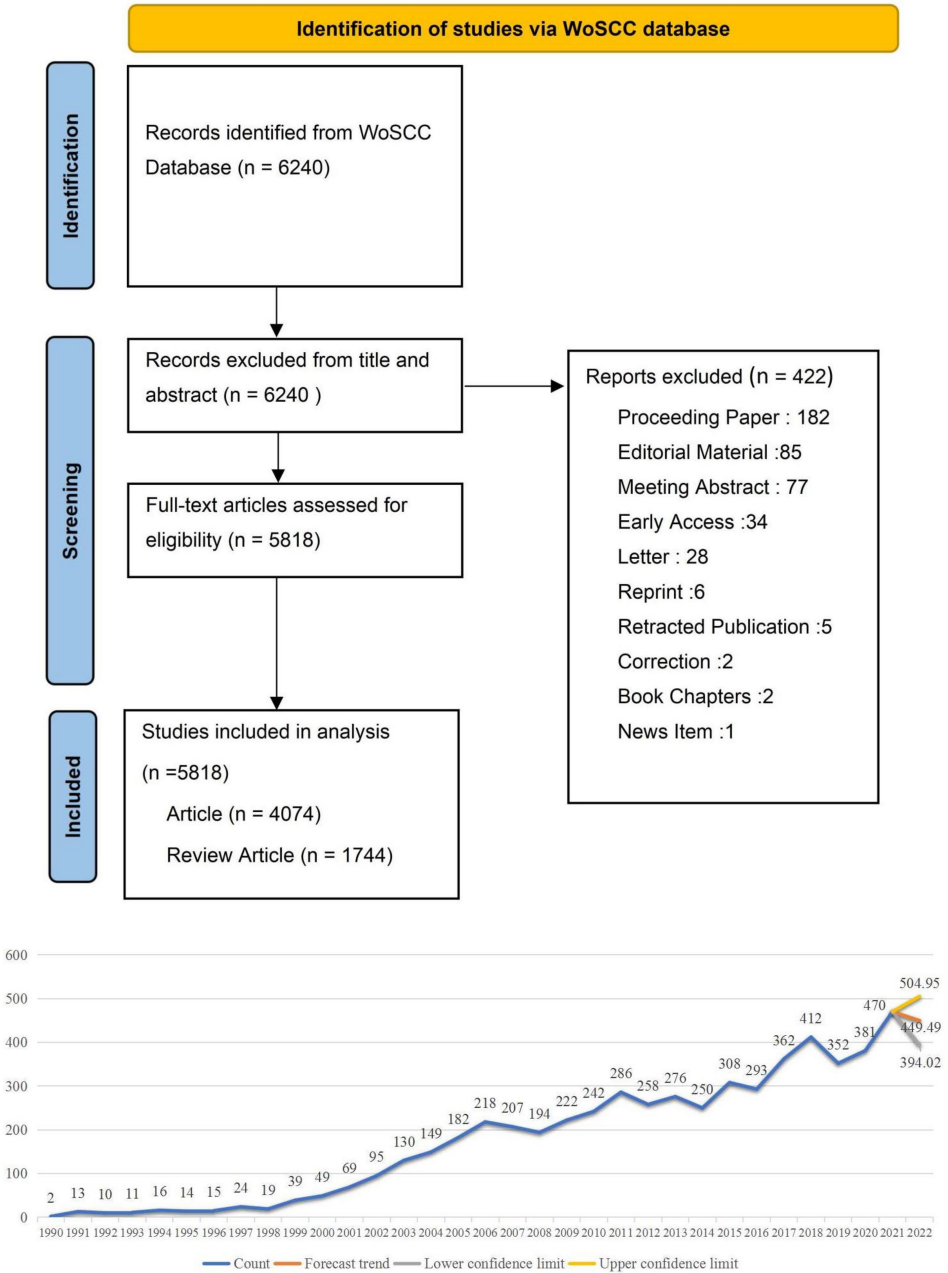
The literature’s prediction graph and the literature’s screening flow chart. This is the trend chart of the distribution and growth of the literature, and we predict that publications in 2022 were between 394 and 504.

The literature data was collected from the Web of Science Core Collection (WoSCC) database through the Science Citation Index Expanded (SCI-E) on August 9, 2022. Our search strategy was: TS = (antiinflammatory or anti-inflammatory or anti-inflammation) AND TS = (coronary heart disease or unstable angina pectoris or angina pectoris or Acute coronary syndrome or heart failure or myocardial infarct) AND PY = (1990–2022) AND LA = (English). Only articles and reviews met the requirement and were included. The literature search was conducted by two authors independently (JL Z and CY J). After data normalization, all documents, including the complete records and cited references, were exported in pure text format. All valid data were imported to VOSviewer and CiteSpace for visual analysis. We analyze the essential characteristics of the literature. Microsoft Excel 2019 was used to predict the growth trend of publications in 2022. Figure 1 shows the literature’s prediction graph and the literature’s screening graph.

VOSviewer is a procedure for building and viewing bibliometric maps (13). It can be used to build author, journal, or keyword maps based on co-occurrence data (14). CiteSpace focuses on analyzing the potential knowledge contained in the scientific literature (15). It can be used to visualize the comprehensive research situation over a certain period and to predict the development trend of the related field (16). CiteSpace has certain advantages in revealing the dynamic development law of the discipline and the research frontier (17). VOSviewer can be selected to draw the knowledge map in presenting the relationship between the subject themes (18).

## Results

### Distribution of literature

A total of 5,818 articles focus on anti-inflammatory studies in CHD. Among the most prolific authors, Libby Peter (USA) was ranked first with 28 articles, followed by Aukrust, Pal (Norway), and Ridker, Paul M (USA), with 27 and 26 articles, respectively. Anker, Stefan D (Germany) and Tousoulis, Dimitris (Greece) were ranked fourth and fifth with 22 and 20 articles, respectively. For the details of all literature, see Supplementary material 1.

In terms of publications, they have now been published in over 1,458 journals. Circulation has published 98 articles on the application of anti-inflammatory in CHD. This was followed by PloS One (84 papers), Atherosclerosis (79 papers), International Journal of Cardiology (68 papers), and Current Pharmaceutical Design (65 papers). Impact Factor (IF) is a quantitative index representing a journal’s impact and general evaluation of international journals’ academic level and publication quality (19). According to the latest impact factor published in 2022, Circulation had the highest impact factor of 39.918, followed by Atherosclerosis with an IF of 6.847.

The literature on anti-inflammatory intervention in CHD has been published in 103 countries and regions. The United States leads the way, with 1,708 publications, followed by China (929 publications), Italy (496 publications), Germany (397 publications), And England (394 publications).

Among the research institutions, 4,959 institutions were involved in the research field. According to the statistical analysis, Harvard University ranked first with 213 articles, followed by the University of California System (149 publications), Brigham and Women’s Hospital (144 publications), University of London (124 publications), and Institut National De La Sante Et De La Recherche Medicale Inserm (111 publications). In conclusion, the top three institutions are all research institutions in the US, where Brigham and Women’s Hospital are affiliated hospitals under Harvard Medical School. The US remains a leader in anti-inflammatory intervention for coronary heart disease, and Harvard University’s system ranks first in various organizations’ publications.

The top 20 most cited references besides the clinical guidelines are shown in Table 1. The top 3 papers were all from the relevant Harvard University team. The first paper comes from Peter Libby’s review in Circulation (Inflammation and atherosclerosis) (20), published in 2002. His review of Inflammation in atherosclerosis was published in Nature in the same year (21). The total citation frequency of the two articles reached 12,576 times. It is clearly stated that inflammation is a therapeutic target in atherosclerosis. In 1997, Paul M. Ridker published in The New England Journal of Medicine (NEJM) in “Inflammation, Aspirin, and the Risk of Cardiovascular Disease in Apparently Healthy Men” (22), suggesting that anti-inflammatory drugs may have a clinical benefit in preventing cardiovascular disease. In 2017, Paul M. Ridker published “Antiinflammatory Therapy with Canakinumab for Atherosclerotic Disease” (23) in NEJM. This randomized, double-blind, placebo-controlled international multi-center clinical trial created the first new era of anti-inflammatory treatment of atherosclerotic diseases and was cited more than 4,000 times.

**Table 1:** The top 20 most cited references.

Title	Authors (top five)	Journal	Year	DOI	Total Citation	Impact Factor
Inflammation and atherosclerosis	Libby, P; Ridker, PM; Maseri, A	CIRCULATION	2002	10.1161/hc0902.104353	5,729	39.9175
Inflammation, aspirin, and the risk of cardiovascular disease in apparently healthy men	Ridker, PM; Cushman, M; Stampfer, MJ; Tracy, RP; Hennekens, CH	NEW ENGLAND JOURNAL OF MEDICINE	1997	10.1056/NEJM199704033361401	4,513	176.0774
Antiinflammatory Therapy with Canakinumab for Atherosclerotic Disease	Ridker, PM; Everett, BM; Thuren, T; MacFadyen, JG; Chang, WH et al.	NEW ENGLAND JOURNAL OF MEDICINE	2017	10.1056/NEJMoa1707914	4,415	176.0774
Chemistry and Biological Activities of Flavonoids: An Overview	Kumar, Shashank; Pandey, Abhay K	SCIENTIFIC WORLD JOURNAL	2013	10.1155/2013/162750	2,163	NA
Chronic subclinical inflammation as part of the insulin resistance syndrome - The Insulin Resistance Atherosclerosis Study (IRAS)	Festa, A; D’Agostino, R; Howard, G; Mykkanen, L; Tracy, RP et al.	CIRCULATION	2000	10.1161/01.CIR.102.1.42	1925	39.9175
Adipose tissue, inflammation, and cardiovascular disease	Berg, AH; Scherer, PE	CIRCULATION RESEARCH	2005	10.1161/01.RES.0000163635.62927.34	1,599	23.213
Recent advances in the relationship between obesity, inflammation, and insulin resistance	Bastard, JP; Maachi, M; Lagathu, C; Kim, MJ; Caron, M et al.	EUROPEAN CYTOKINE NETWORK	2006	PMID: 16613757	1,463	3.45
Interleukin-1 in the pathogenesis and treatment of inflammatory diseases	Dinarello, Charles A.	BLOOD	2011	10.1182/blood-2010-07-273,417	1,432	25.476
Review of the biology of quercetin and related bioflavonoids	Formica, JV; Regelson, W	FOOD AND CHEMICAL TOXICOLOGY	1995	10.1016/0278-6,915(95)00077-1	1,404	5.572
Risk of cardiovascular events associated with selective COX-2 inhibitors	Mukherjee, D; Nissen, SE; Topol, EJ	JAMA-JOURNAL OF THE AMERICAN MEDICAL ASSOCIATION	2001	10.1001/jama.286.8.954	1,375	157.335
Omega-3 fatty acids in inflammation and autoimmune diseases	Simopoulos, AP	JOURNAL OF THE AMERICAN COLLEGE OF NUTRITION	2002	10.1080/07315724.2002.10719248	1,375	3.571
Effect of statin therapy on C-reactive protein levels - The Pravastatin Inflammation/CRP Evaluation (PRINCE): A randomized trial and cohort study	Albert, MA; Danielson, E; Rifai, N; Ridker, PM	JAMA-JOURNAL OF THE AMERICAN MEDICAL ASSOCIATION	2001	10.1001/jama.286.1.64	1,374	157.335
Associations of elevated interleukin-6 and C-reactive protein levels with mortality in the elderly	Harris, TB; Ferrucci, L; Tracy, RP; Corti, MC; Wacholder, S et al.	AMERICAN JOURNAL OF MEDICINE	1999	10.1016/S0002-9343(99)00066-2	1,215	5.928
Bioactive compounds in foods: Their role in the prevention of cardiovascular disease and cancer	Kris-Etherton, PM; Hecker, KD; Bonanome, A; Coval, SM; Binkoski, AE et al.	AMERICAN JOURNAL OF MEDICINE	2002	10.1016/S0002-9343(01)00995-0	1,210	5.928
Vascular and upper gastrointestinal effects of non-steroidal anti-inflammatory drugs: meta-analyses of individual participant data from randomised trials	Bhala, N; Emberson, J; Merhi, A; Abramson, S; Arber, N et al.	LANCET	2013	10.1016/S0140-6736(13)60900-9	1,103	202.7275
Antiinflammatory properties of HDL	Barter, PJ; Nicholls, S; Rye, KA; Anantharamaiah, GM; Navab, M et al.	CIRCULATION RESEARCH	2004	10.1161/01.RES.0000146094.59640.13	1,024	23.213
Flavonoids: Old and new aspects of a class of natural therapeutic drugs	Di Carlo, G; Mascolo, N; Izzo, AA; Capasso, F	LIFE SCIENCES	1999	10.1016/S0024-3205(99)00120-4	1,010	6.78
The Biological Basis for Cardiac Repair After Myocardial Infarction From Inflammation to Fibrosis	Prabhu, Sumanth D; Frangogiannis, Nikolaos G	CIRCULATION RESEARCH	2016	10.1161/CIRCRESAHA.116.303577	958	23.213
Update on uses and properties of Citrus flavonolds: New findings in anticancer, cardiovascular, and anti-inflammatory activity	Benavente-Garcia, O; Castillo, J	JOURNAL OF AGRICULTURAL AND FOOD CHEMISTRY	2008	10.1021/jf8006568	808	5.895

The Scientific World Journal was deselected from SCIE on September 15th, 2014. All the impact factors come from the latest Journal Citation Report published by Clarivate in 2021.

### Cooperative analysis

Big data reveals that cooperation between high-level academics or research institutions can produce more effective results (24). The analysis of literature authors and their cooperative network is conducive to grasping the cooperation between high-yielding authors and academic groups in this research field (25, 26).

**Figure 2:**
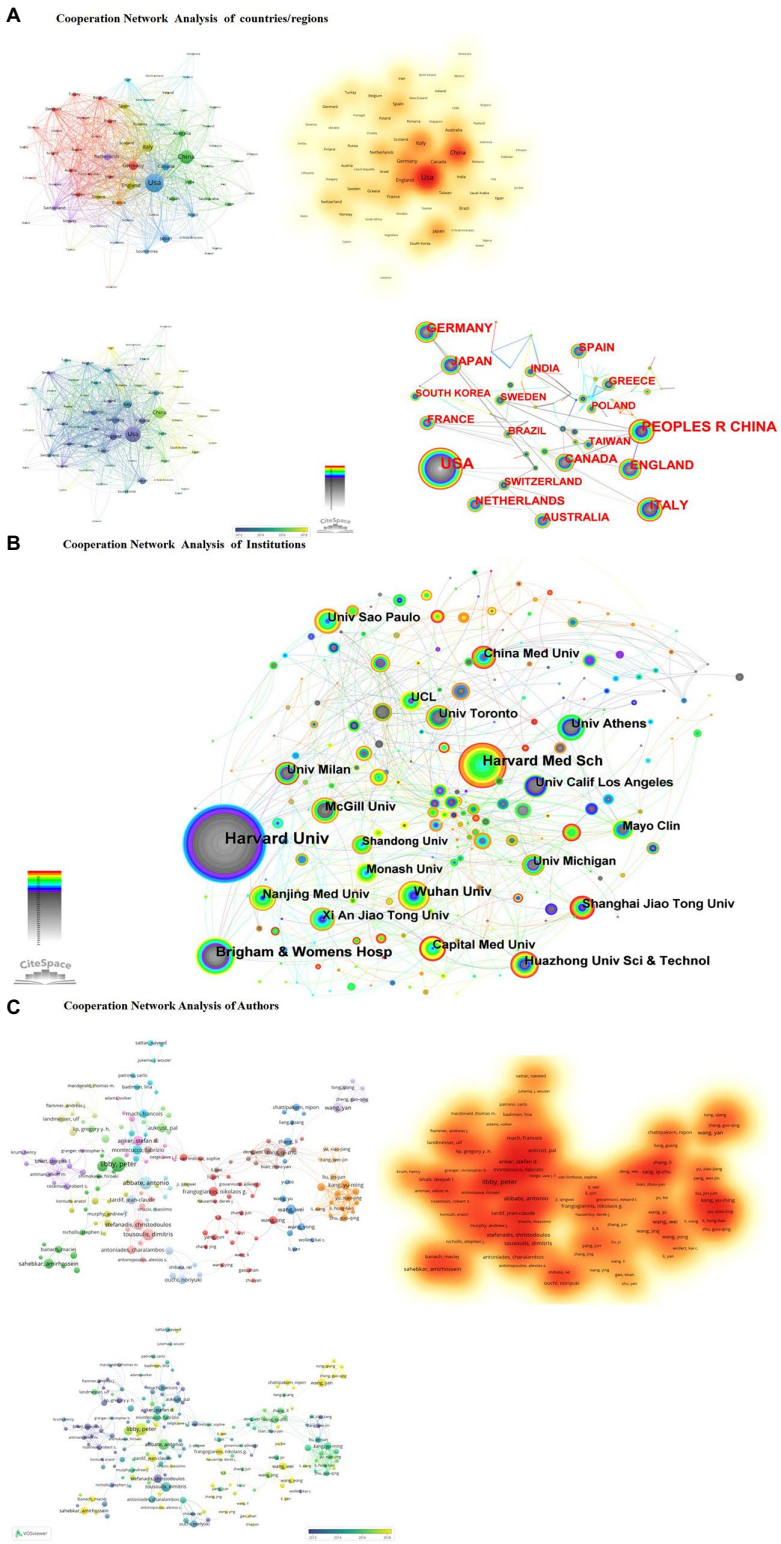
**(A)** Cooperation network analysis of countries/regions. **(B)** Cooperation network analysis of institutions. **(C)** Cooperation network analysis of authors.

In the national cooperation analysis, there are three main trends. First, the United States and China as the central core cluster. Second, it has three obvious geographical advantages, the American continent research cluster, the research cluster of European countries, and the East Asian research cluster, and third, there are spatio-temporal change trends. Before 2014, mainly in Europe and America and other developed countries, after 2014, China and developing countries in Asia began to emerge (Figure 2A).

In the analysis of institutional cooperation, from the perspective of the frequency of institutional collaboration, the Harvard University system was dominated in the early stage. Harvard Medical School, Brigham & Women’s Hosp participated; after 2010, the University of Amsterdam, Wuhan University, and Nanjing Medical University participated in the research. China followed closely in the study, with a small-scale but systematic cluster of research institutions (Figure 2B).

In the author’s cooperation analysis, there are two apparent characteristics; Developed countries such as Europe and the United States have presented a research pattern of the cooperative cluster formed by a group of Harvard University experts led by Peter Libby and Paul M. Ridker. Developing countries, represented by Chinese experts’ concentrated research, including Qizhu Tang’s (27–33) and Wei Wang’s (34–39) team, have characteristics that emphasize the role of traditional medicine and natural products in the anti-inflammatory treatment of CHD (Figure 2C).

### Journal analysis

Academic journals serve as a vehicle for disseminating disciplinary knowledge, and the relationship between the journals cited in each discipline can reflect the flow of knowledge between journals. Journal coupling analysis refers to the reference situation where two pieces of literature are cited together (40), i.e., if two articles cite the same literature simultaneously, there is a coupling relationship between the two articles. Coupling analysis connects journals from the perspective of knowledge absorption, exploring the academic classification, determining the core or peripheral status of periodicals, the disciplinary nature of journals, and the degree of correlation among various domains. Journal co-citation refers to the co-citation of two articles that appear in the reference list of a third cited article (41). Journal co-citation analysis examines associations between journals from the perspective of knowledge output, and looking at co-citation relationships between journals provides a glimpse into the scholarly communication patterns of disciplinary research.

#### Journal co-citation analysis

**Figure 3:**
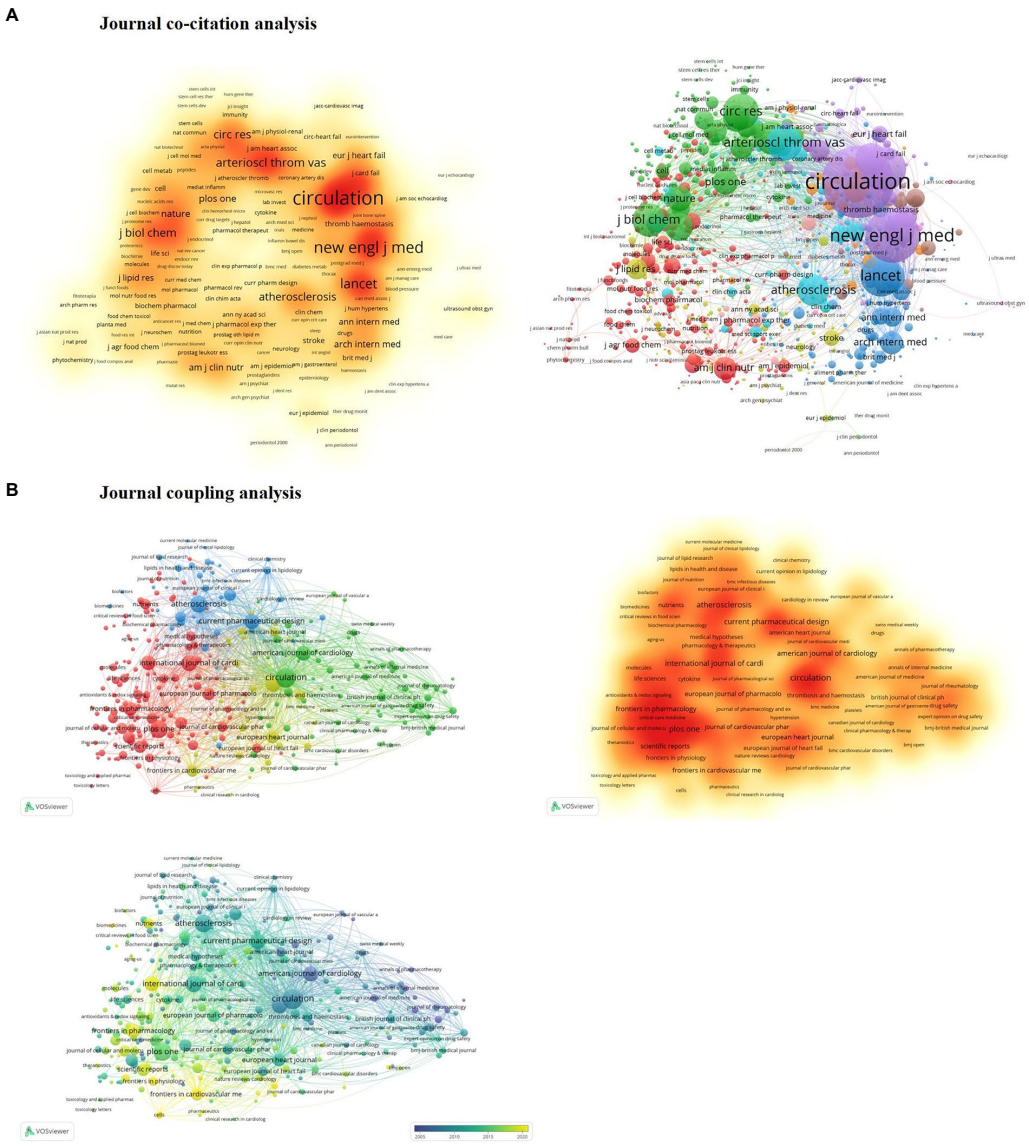
**(A)** Journal co-citation analysis. **(B)** Journal coupling analysis.

All journals had a total of 346,140 citations. From the total citations of the journals, Circulation ranked first with 21,817 citations, followed by NEJM. NEJM published only nine publications, but these nine references’ total citations reached 14,256 times. Lancet published 12 related articles with a total frequency of 6,285 citations. Among the latest journal impact factor, the Circulation score was 39.918, ranking second in Cardiology and Cardiovascular Medicine. The lancet score was 202.731, and the NEJM impact factor was indexed third in the Medicine disciplines, at 176.079. Although the number of publications is low, internationally renowned academic journals greatly influence co-citation (Figure 3A).

#### Journal coupling analysis

The journal coupling method can be used to analyze the relationship between journals, help journal classification and explore the internal knowledge structure of the disciplines. The larger node also shows the more significant influence of the journals. We screened journals with a minimum citation count of five times, yielding 294 publications. In the journal coupling analysis, we can find that; Early years focus on the association of traditional publishing journals. Moving on to open access journals after 2015. With an apparent trend, early years as Circulation, American Journal of Cardiology, Atherosclerosis, International Journal of Cardiology, Current Pharmaceutical Design’s multiple non-open access journals, as the leading core group. Journal coupling density maps formed at later stages, including coupled clusters with open access journals (Figure 3B).

### Cluster analysis of keywords

Keywords are the values used to identify specific data items in the literature, mainly to briefly and accurately describe the article’s topic, and are primarily used for indexing or cataloging (42). Therefore, we can understand publications’ characteristics and evolution trends by analyzing the changes in keywords.

**Figure 4:**
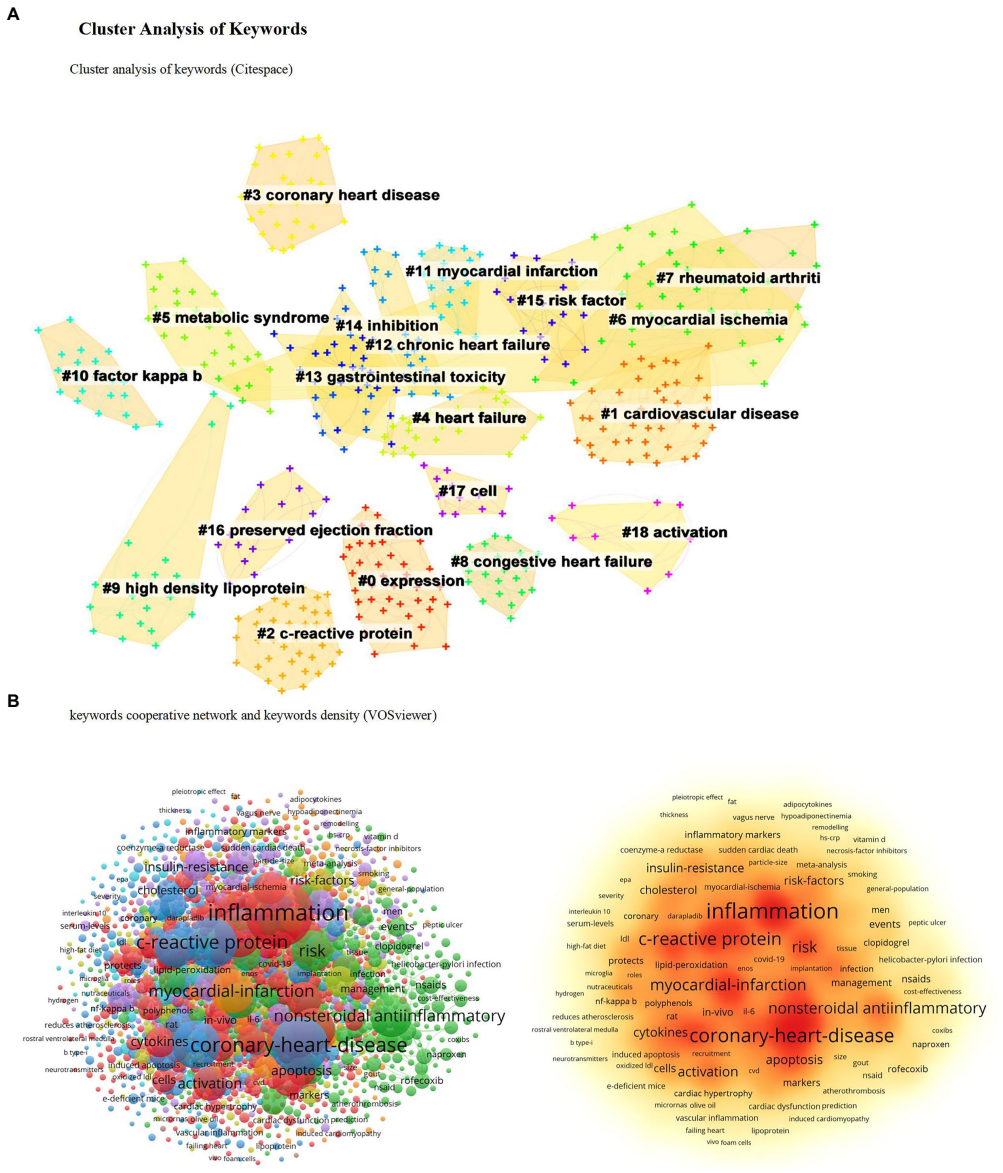
Cluster analysis of keywords. **(A)** Cluster analysis of keywords (Citespace). **(B)** Keywords cooperative network and keywords density (VOSviewer).

A keyword merged coexistence network with 459 nodes and 5,024 links were constructed using CiteSpace. The parameters of the software are set as follows. Time slicing: from 1990 to 2022, 1 year per slice. Node types: reference. Selection criteria: select the top 50 levels of most occurred items from each slice. Cluster analysis was performed based on these keywords

and the results are shown in (Figure 4A). The smaller the cluster tag number

the larger the cluster size. According to the cluster results analysis

the critical clusters are coronary heart disease

c-reactive protein

cardiovascular disease

myocardial infarction

rheumatoid arthritis

and gastrointestinal toxicity. According to the VOSviewer analysis of the keyword cooperative network and keyword density

the top five core keywords are inflammation

C-reactive protein

coronary heart disease

nonsteroidal antiinflammatory

and myocardial infarction (Figure 4B)

### Keywords and references with the strongest citation bursts

**Figure 5:**
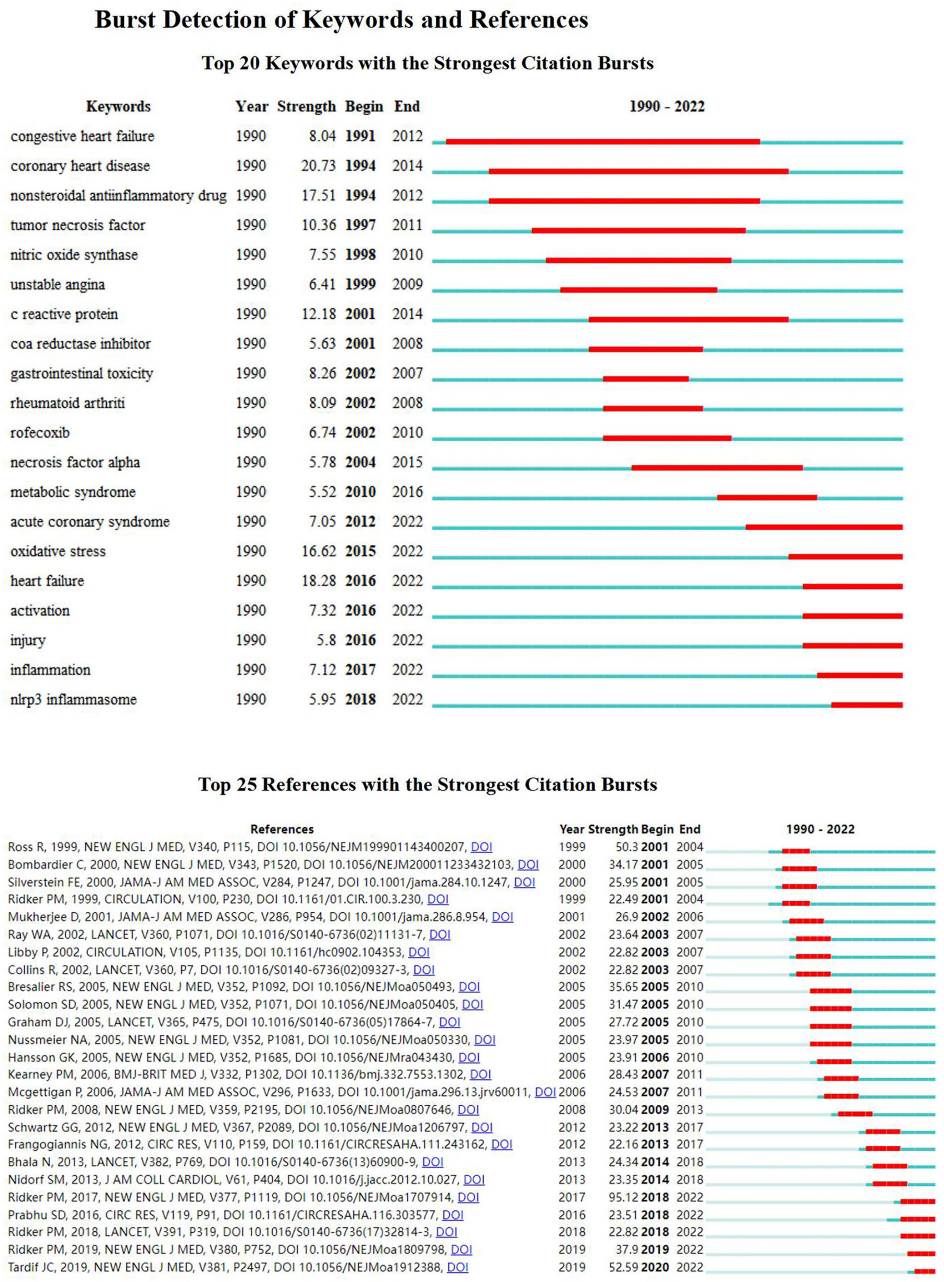
Burst detection of keywords and references. Top 20 keywords with the strongest citation bursts. Top 25 references with the strongest citation bursts.

CiteSpace provides burst detection that can perceive significant changes in references and keywords over a certain period (43). We screened the top 20 keywords and the 25 references according to burst intensity (Figure 5). The strongest keywords were “coronary heart disease (20.73),” and most recently “heart failure (18.28),” nonsteroidal anti-inflammatory drug (17.51), oxidative stress (16.62), c reactive protein (12.18), tumor necrosis factor (10.36). In addition, the Nlrp3 inflammasome in the last 2 years strongly references the sudden hot spot, reflecting the current hot spot trend. Moreover, the most cited citation burst is “Ridker PM, 2017 (95.12),” ranked by citation time. The top five are Tardif JC, 2019 (52.59), Ridker PM, 2019 (37.9), Ridker PM, 2018 (22.82), Prabhu SD, 2016 (23.51), and Ridker PM, 2017 (95.12).

The strongest keywords were “coronary heart disease (20.73),” and most recently “heart failure (18.28), nonsteroidal anti-inflammatory drug (NSAIDs) (17.51), oxidative stress (16.62), c reactive protein (12.18), and tumor necrosis factor (10.36). In addition, the Nlrp3 inflammasome in the last 2 years is a strong reference to the sudden hot spot, reflecting the current hot spot trend.

Moreover, the most cited citation burst is “Ridker PM, 2017 (95.12),” ranked by citation time; the top five are Tardif JC, 2019 (52.59), Ridker PM, 2019 (37.9), Ridker PM, 2018 (22.82), Prabhu SD, 2016 (23.51) and Ridker PM, 2017 (95.12).

### Cluster analysis of reference co-citation

**Figure 6:**
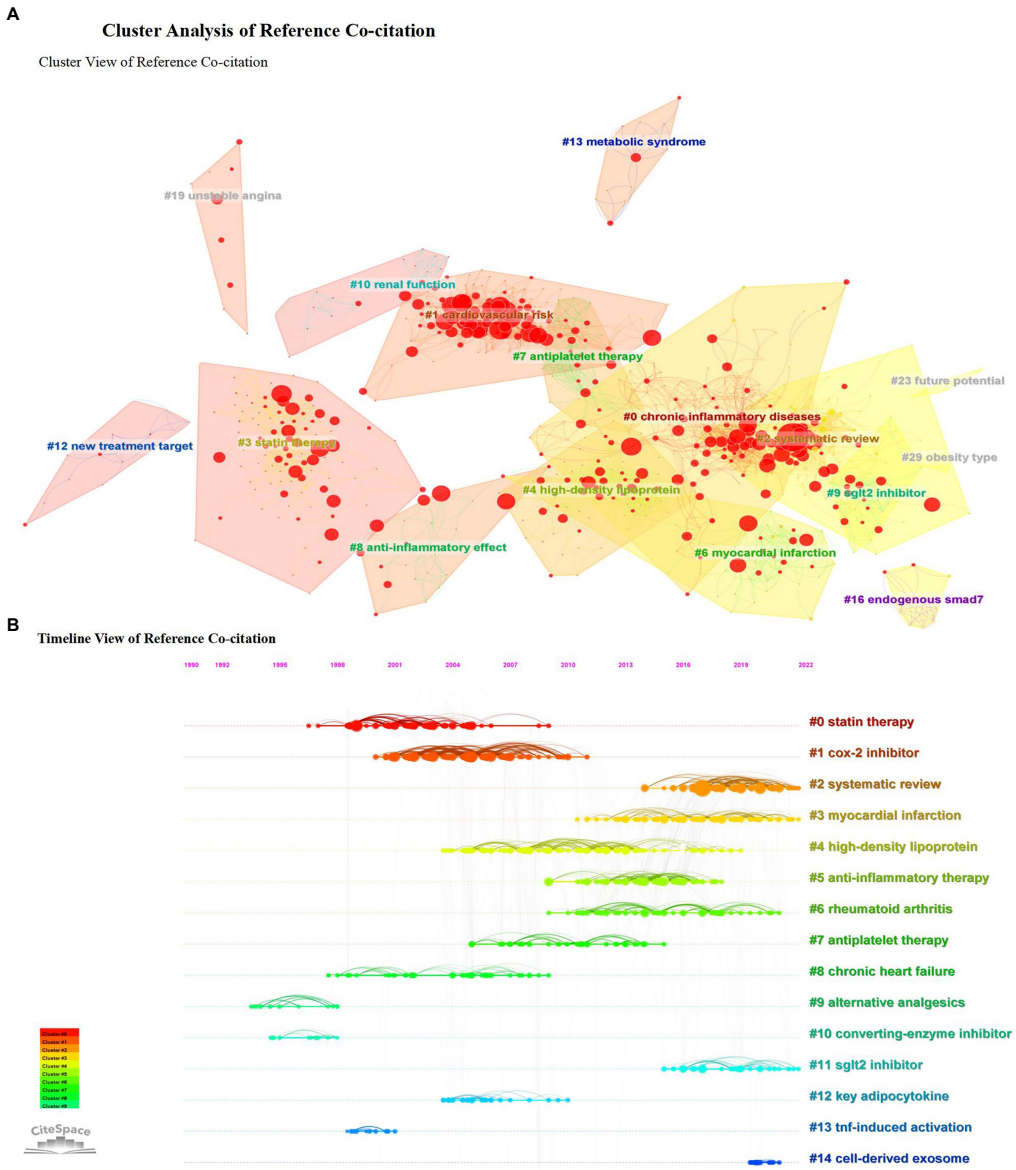
Cluster analysis of reference co-citation. **(A)** Cluster view of reference co-citation. **(B)** Timeline view of reference co-citation.

According to the analysis of study topics, reference co-citation clustering is a superior function of CiteSpace, enabling us to study issues and hotspot trends comprehensively. Each cluster is considered to represent a hot frontier of research. Therefore, we used CiteSpace to describe the cluster view (Figure 6A) and the timeline view (Figure 6B) of the reference co-citations to analyze the trend of anti-inflammatory applications in CHD. The parameters of the software are set as follows. The top 50 papers (TOP = 50) references of each time section were also extracted to construct a literature co-citation network. Contains 4,146 lines and 964 nodes. The analysis yielded a cluster modularity value of 0.799 (Q value), reflecting that the clustering is apparent.

The clustering results jointly cited by references give us some hints: early attention focus on the anti-inflammatory effects of statins (44–46), recent hot topics pay more attention to the study of combined diseases and comorbidities, to explore the common mechanism of anti-inflammation in coronary heart disease and other significant chronic non-communicable complex diseases such as diabetes and obesity (47–49). In addition, the application of new anti-inflammatory drugs is also one of the hot concerns (50–53).

## Discussion

Coronary heart disease has become the highest mortality disease in the world. The number of cardiovascular diseases in China is 290 million, containing 11 million coronary heart diseases, and morbidity trends are increasing yearly (54, 55). With the development of research, it found that inflammation plays an important role in the development of CHD. The inflammatory response is an essential mechanism of CHD and significantly impacts the progression of coronary atherosclerotic plaque and adverse cardiovascular events (56, 57). The inflammatory response accelerates the formation of atherosclerotic plaques. Some inflammatory factors reduce the tensile strength of the plaque fiber cap and increase the necrotic lipid core, leading to damaged endothelium and plaque rupture (58–60). Meanwhile, anti-inflammatory treatment has been proven effective in the secondary prevention of coronary heart disease, reducing acute coronary events and improving the prognosis (61, 62).

The number of anti-inflammatory interventions in CHD-related studies has generally increased over the past 30 years. It shows that inflammation, as a critical pathological change during atherosclerosis progression, is attracting increasing attention from researchers (63). Early studies confirmed the correlation between CRP and inflammatory mechanisms in CHD. Acute phase CRP has been shown to reflect systemic and possible vascular inflammation and to predict future cardiovascular events in asymptomatic individuals. In addition, CRP promotes the release of TNF-α, IL-1β, and IL-6 from macrophages and foam cells in the neointima (64). The release of these pro-inflammatory factors promotes atherosclerosis and recruits early monocytes and lymphocytes in the intima (65). One study (66) showed that serum hs-CRP levels were significantly higher in patients with moderate and severe arterial stenosis than in patients with mild stenosis. A panel of experts from the Centers for Disease Control and Prevention and the American Heart Association (67) has recommended hs-CRP as the clinical test with the highest reliability for assessing and predicting the risk of cardiovascular disease. Hs-CRP has become an independent predictor of atherosclerosis and cardiovascular disease, classifying individuals into three risk categories based on hs-CRP levels. Low <1 mg/l; medium 1–3 mg/l; and high >3 mg/l. Each standard deviation increase in hs-CRP concentration increases the risk by 45%, so the level of hs-CRP can be applied to distinguish low-risk from high-risk for atherosclerosis and cardiovascular disease.

In addition, there is a class of clusters focused on rheumatoid arthritis, which is not exploring the comorbid mechanisms of RA and CHD, but instead uses anti-inflammatory drugs to treat rheumatoid arthritis. Recent studies have focused on mechanistic intervention in cardiovascular disease with the anti-inflammatory drugs allopurinol (68, 69) and colchicine (70–74), promising agents for intervention in cardiovascular disease (75). Moreover, there have been concentrated studies on gastrointestinal toxicity of NSAIDs (76–78), such as targeting gastrointestinal side effects of anti-inflammatory medications COX-2 inhibitors (79, 80), focusing on the drug’s safety, and preventing adverse events (81, 82).

According to the time trend analysis of the keyword strongest burst, from the late 1990s, the anti-inflammatory application of traditional indicators (tumor necrosis factor, CRP) was concerned. Around 2010, research focused on NSAIDs’ use in CHD, including the gastrointestinal toxicity of NSAIDs (77, 83). In the past 5 years, more attention has been paid to the mechanism of the inflammasome in coronary heart disease (84), and the NLRP3 inflammasome-driven IL-1 release has led to atherosclerotic progression and accelerated vascular inflammatory response (85, 86). The research on inflammasome may become one of the research hotspots for future anti-inflammatory interventions in CHD. Although hs-CRP can be used clinically as a biomarker for risk prediction, and high levels of hs-CRP are associated with adverse cardiovascular outcomes after acute coronary syndrome (ACS) (87), most mechanistic studies suggest that CRP itself is unlikely to be an ideal target for specific anti-inflammatory therapies (88). Upstream movement of the inflammatory cascade from CRP to IL-6 to IL-1 offers new therapeutic opportunities for atherosclerosis protection (89). IL-1β, a critical pro-inflammatory cytokine, is involved in various autoimmune inflammatory responses (90). The level of IL-1β is synergistically regulated by Toll-like receptors (TLRs) and Nod-like receptors (NLRs). Activation of TLRs induces the synthesis of precursor IL-1β and precursor interleukin-18, and the activation of NLRs induces assembly in the host cell cytoplasm to form a multimeric protein complex, the inflammasome. The inflammasome is central to the production of IL-1β and IL-18.

The NLRP3 inflammasome/IL-1β signaling pathway plays an important role in the development of AS. Caspase-1, IL-1β, and IL-18, related components of the NLRP3 inflammasome signaling pathway, were highly expressed in atherosclerotic plaques, and the expression levels were higher in vulnerable plaques than in stable plaques (91), indicating that the NLRP3 inflammasome pathway plays an essential role in the development of AS and affects plaque stability. Cytokine release inhibitory drug 3 (MCC 950), a selective inhibitor of the NLRP3 inflammasome (92), was shown to reduce the maximum stenosis significantly, mean plaque size and volume, minimize macrophage aggregation and inflammatory factor levels, and increase plaque stability in the aorta of apoE−/− mice (93). MCC 950 blocks the NLRP3 inflammasome/IL-1β signaling pathway from upstream, reducing the production of not only IL-1β but also inflammatory factors such as IL-1α and IL-18 simultaneously (94). Theoretically, it is a safer and more effective therapeutic measure than the anti-IL-1β monoclonal antibody canakinumab and has good research prospects. Targeted modulation of the NLRP3 inflammasome/IL-1β is expected to be one of the hot studies for future anti-inflammatory interventions in the prevention and treatment of CHD (95, 96).

Similar results were shown according to the timeline of the research topic. The early 90s focused on traditional mechanisms such as tumor necrosis factor (97, 98) and the mechanism of a nuclear transcription factor (99–101) in atherosclerosis. Several cytokines, including TNF-α, TGF-β, and different interleukins, are involved in developing various inflammatory cardiac pathologies (102, 103). It was found that the combined action of the NF-κB signaling pathway and IL-23/IL-17 inflammatory axis allows IL-1β and TNF-α to accumulate in macrophage foam cells and inflammatory responses, both of which are involved in the pathological development of CHD and related diseases (104).

Around 2000, attention was paid to the anti-inflammatory effects of statins in patients by regulating CRP in CHD (105–108). Despite aggressive statin therapy, publications (109, 110) show that inflammation may be an important driver of residual cardiovascular risk in coronary artery disease. Due to the inability of lipid-lowering to slow the progression of atherosclerosis completely, the identification of inflammatory biomarkers as independent risk factors for cardiovascular disease events has facilitated trials using anti-inflammatory strategies to treat atherosclerosis (111, 112). Since 2010, the focus has been on the mechanisms of NSAIDs in CHD, especially pilot studies using anti-rheumatoid arthritis drugs such as colchicine (62, 113) and methotrexate (114) to intervene in CHD and modulate the level of inflammation. Different from the keyword burst, in the last 5 years, on the one hand, attention has been paid to clinical studies, and the integration of evidence has been carried out to evaluate the evidence systematically (115, 116). On the other hand, studies on the potential association between macrophages and anti-inflammatory have been carried out, which will help drive the formation of new therapies (117–119). In addition, the anti-inflammatory efficacy of fish oil in cardiovascular diseases is included as an area of exploration in nutrition (120–122).

The references with the strongest citation bursts revealed that recent attention has focused on the anti-inflammatory clinical studies of Canakinumab and colchicine in cardiovascular diseases (61, 123–126). The CANTOS study (23) enrolled 10,061 patients from 39 countries with myocardial infarction combined with elevated hs-CRP (>2 mg/l). Canakinumab is a selective, high-affinity, fully humanized monoclonal antibody that targets the inhibition of interleukin-1β (IL-1β). The study showed that canakinumab could further reduce adverse cardiovascular events with myocardial infarction on top of lipid-lowering drug therapy (57, 127, 128). The CANTOS confirmed the clinical importance of the pro-atherosclerotic of IL-1β and identified the IL-1 to IL-6 to CRP inflammatory pathway as a central target for atherosclerotic protection. These data support further drug discovery of atherosclerotic thrombosis therapies targeting IL-18 or IL-6. Due to the role of IL-1β in promoting various pro-inflammatory factors previously, the search for signaling pathways upstream of IL-1β (e.g., NLRP3 inflammasome inhibitors) and possible inflammation targets for intervention has become a hot topic of current research (129). In a follow-up study (130) of 4,833 CANTOS participants, inhibition of the IL-6 signaling pathway was associated with reducing cardiovascular events and all-cause mortality. IL-6 is involved in the pathogenesis of multiple inflammatory diseases, and plasma IL-6 levels strongly predict future vascular events independent of traditional risk factors (131). The results of this study also suggest that lower IL-6 ratios may lead to a lower proportion of cardiovascular events.

Allopurinol is commonly used as a first-line agent to lower serum uric acid and prevent acute attacks in patients with gout, and cardiovascular benefits have also been reported (68, 69). A population-based case–control study (132) found that allopurinol was associated with a lower risk of non-fatal acute myocardial infarction and that the longer patients took the drug, the greater the reduction in infarction risk, suggesting additional cardiovascular protection. In a recent ALL-HEART trial conducted in the United Kingdom (133), allopurinol combined with conventional therapy did not improve cardiovascular outcomes (non-fatal myocardial infarction, non-fatal stroke, or cardiovascular death) in patients with ischemic heart disease. Therefore, the trial shows that allopurinol may not be recommended for the secondary prevention of cardiovascular events in patients with ischemic heart disease. New evidence for the cardiovascular benefit of allopurinol remains to be further investigated.

Colchicine is widely used in clinical practice for gout (113, 134, 135). The LoDoCo (136) study suggested that colchicine reduced the relative risk of the primary endpoint event (acute coronary syndrome, out-of-hospital cardiac arrest, or non-cardiogenic embolic ischemic stroke) by 67% in 532 patients with stable coronary artery disease treated with low-dose colchicine (0.5 mg/day) (HR, 0.33; 95% CI, 0.18 to 0.59; *p* < 0.001). The COLCOT (137) published in 2019 is a large randomized controlled trial (RCT) evaluating the effect of the colchicine group (0.5 mg/day) on recurrent cardiovascular events in patients who had a myocardial infarction within 30 days. The results showed a significant 23% (HR, 0.77; 95% CI, 0.61 to 0.96; *p* = 0.02) reduction in the risk of the primary endpoint event (including cardiovascular death, cardiac arrest, non-fatal myocardial infarction, non-fatal stroke, and urgent revascularization due to angina). The results of a CT coronary angiography study (138) of colchicine intervention in ACS showed that low-dose colchicine treatment was effective in modifying coronary plaques with ACS and that the anti-inflammatory properties of colchicine may drive the improvement in plaque morphology. Colchicine may be beneficial as an additional secondary prevention drug in patients post-ACS. Recent meta-analyses (139–142) have shown that colchicine positively reduces the incidence of MACE, MI, stroke, and revascularization and decreases cardiovascular events, inflammatory markers, hs-CRP, and IL-6 in patients with coronary artery disease. But with a higher incidence of gastrointestinal distress and no effect on all-cause mortality. In a recent Australian study (143), patients with ACS were treated with colchicine 0.5 mg/d twice daily for the first month, then 0.5 mg daily for 11 months, and after 1 year of follow-up, there was no significant difference in the primary adverse event composite endpoint in patients taking colchicine compared to the placebo. After 2 years follow-up (144), the primary adverse cardiovascular event endpoint incidence was significantly lower. This sustained effect may be attributed to colchicine’s anti-inflammatory and plaque-modulating properties, reducing the potential development of high-risk plaque volume and ischemic complications. Since the drug was only used for 12 months, the results sustained over 2 years suggest that colchicine may have a legacy effect. Several studies with different trial designs, including colchicine dose, the timing of administration, and the different endpoint events, may have influenced the trial results. More comprehensive and in-depth studies are needed to provide definitive evidence for the clinical use of colchicine. In summary, these findings initially suggest an opportunity to reduce the burden of coronary heart disease in patients using either drug targeting IL-1β or other inflammatory inhibitory pathways (145). Future trials of other new anti-inflammatory agents may help to understand the role of anti-inflammation in the prevention of severe cardiovascular disease events in high-risk patients. We list recent ongoing clinical studies of anti-inflammatory interventions for coronary artery disease being recruited by ClinicalTrials.gov in Supplementary material 2.

In conclusion, integrating the burst of the keywords and the thematic timeline, the current research is focused on the mechanism of anti-inflammation and anti-inflammatory drugs in CHD, and the association between inflammatory vesicles NLRP3 levels and coronary heart disease risk is one of the hot topics (84). In addition, supplementing dietary nutrients and trace elements (146), including omega-3 fatty acids (147–149), provides a nutritional perspective for anti-inflammatory intervention in cardiovascular disease. A recent study has also found (150) that ferroptosis plays a crucial role in the development of CHD and that antioxidants may be the most promising inhibitors of ferroptosis in widespread use. Ferroptosis inhibition is a good option for treating CHD. Moreover, smartphone-based applications (151) for health management in the anti-inflammatory treatment of coronary heart disease can help bridge the digital divide and may be one of the next hot spots in the post-COVID-19 era.

To the best of our knowledge, this is the first study summarizing the research progress on anti-inflammatory in CHD studies by bibliometric analysis, intuitively presenting contributors, collaboration networks, research hotspots, and development prospects through visualization. This paper analyzes the research trends and hot spots of anti-inflammation in CHD. Researchers can refer to the research trends and grasp the current research hotspots. Meanwhile, researchers can adjust the study design according to the research hotspots to make the study more innovative and feasible. The future can focus on three main points to concentrate on exploration.

1. Inflammatory mechanisms in cardiovascular diseases. Recent studies demonstrating that anti-inflammatory interventions can prevent atherosclerotic complications have only scratched the surface of the potential for developing new therapies. Targeting IL-1β highlights the inflammatory vesicle pathway as a promising avenue for further therapeutic interventions (152). NLRP3 inflammasome and the downstream cytokines IL-1β (153), IL-18 (154, 155), and IL-6 (156) are attractive candidates for intervention.
2. Clinical studies of targeted anti-inflammation. The inflammatory process affects all stages of the atherosclerotic plaque life cycle and is a well-established target for intervention in the disease. CANTOS confirms that IL-1β is a tempting target for anti-inflammatory therapy in CHD and suggests that patients with residual inflammation risk (RIR) are the main population for anti-inflammatory therapy (157). It shows that future anti-inflammatory treatment should move from macro to precision anti-inflammatory therapeutics (158–162). Macrophages are involved in the entire process of atherosclerosis formation, progression, and regression (163). They are the primary inflammatory cells involved in atherosclerosis, and their retention within the arterial tubes is necessary for atherosclerosis (164). The accumulation and functional activation of macrophages in the subintima and the secretion of various pro-inflammatory factors lead to the progression of plaques into chronic complex lesions. Therapeutic strategies that promote the conversion of the macrophage phenotype to an anti-inflammatory phenotype may benefit the prognosis of atherosclerotic cardiovascular disease. Several new anti-inflammatory and anti-cytokine agents, including but not limited to direct upstream inhibitors of the NLRP3 inflammasome, and natural inhibitors of IL-6, can be expected to be used in atherosclerosis by targeting the NLRP3, IL-1, IL-6 to CRP pathway. The way to incorporate these anti-inflammatory agents in practice is long and challenging. Still, discovering potential inflammatory targets demonstrates the importance of addressing this factor for CHD risk prevention.
3. Natural products as anti-inflammatory supplements. Recently, anti-inflammatory nutritional supplements, including fish oil, have attracted widespread attention. Inflammation-induced by dietary components is usually chronic and often caused by alterations in the intestinal flora (165). Therefore, microbial-targeted therapies, such as probiotics, prebiotics, and synbiotics, have great potential in systemic inflammatory diseases. Besides, there is a class of studies (166–168) focusing on the mechanisms of action of natural products in anti-inflammatory intervention for CHD. Evidence suggests that medical plants’ phenolics, and saponins could reduce inflammatory reactions. In addition, various nutritional components within plant flavonoids (169–172), antioxidant vitamins (173–176), and fruit polyphenols (177–179) have the potential to modulate susceptibility to chronic inflammation.

However, it is important to consider that our study still has these limitations. First, this study only contains literature written in English and no literature in other languages, which may bias the study results. Second, we only retrieved data from the WoSCC database, which may lead to incomplete literature collection. Still, it is noteworthy that the academic community recognizes WoSCC as one of the most authoritative literature data platforms covering most studies. Third, the parameter setting and analysis methods of CiteSpace software lack systematic standards, which may lead to discrepancies in the results, so we used CiteSpace combined VOSviewer to achieve a complete visual presentation. Furthermore, articles published in the past 2 years with high impact factor publications were less cited. Therefore, some recently published papers with high quality should have been included in the analysis of highly cited articles.

## Conclusion

This study systematically analyzes the global research results of anti-inflammatory intervention for CHD over the past 30 years. It explores the already published papers’ hotspots, frontiers, and trends. Overall, the literature on anti-inflammatory intervention in coronary heart disease has increased yearly. It is expected that nearly 500 articles will be published in the whole year 2022. Further investigation into the interaction between inflammatory mechanisms may be a future research direction, and the treatment’s screening and efficacy evaluation of anti-inflammatory drugs is the focus. Our results summarize the current status of the study and are of great significance for future clinicians and researchers to condense their research directions.

## Data availability statement

The original contributions presented in the study are included in the article/Supplementary material, further inquiries can be directed to the corresponding authors.

## Author contributions

JH and HT designed this study. JZ and CJ collected all the articles and wrote the manuscript. CJ carried out software operation and figure drawing. XZ contributed to the final version. All authors contributed to the article and approved the submitted version.

## Funding

The work is supported by the National Key R&D Program of China (2019YFC1708501), the National Natural Science Foundation of China (82074316), and the scientific and technological innovation project of Foshan City in the field of traditional Chinese medicine (2020001005585) and the Fundamental Research Funds for the Central Public Welfare Research Institutes (YZ-202142 and YZ-202218).

## Conflict of interest

The authors declare that the research was conducted in the absence of any commercial or financial relationships that could be construed as a potential conflict of interest.

## Publisher’s note

All claims expressed in this article are solely those of the authors and do not necessarily represent those of their affiliated organizations, or those of the publisher, the editors and the reviewers. Any product that may be evaluated in this article, or claim that may be made by its manufacturer, is not guaranteed or endorsed by the publisher.
