# Supplementary material for: Frontiers and hotspots evolution in anti-inflammatory studies for coronary heart disease: A bibliometric analysis of 1990–2022
Source: Front Cardiovasc Med. 2023 Feb 16;10:1038738. doi: 10.3389/fcvm.2023.1038738 (PMC9978200; doi:10.3389/fcvm.2023.1038738)
Supplement: Supplementary file 2 [file Data_Sheet_1.PDF]

ClinicalTrials.gov Search Results 11/13/2022

	NCT Number	Title	Other Names	Status	Conditions	Interventions	Characteristics	Population	Sponsor/ Collaborators	Funder Type	Dates	Locations
1	NCT05347316	Colchicine Effect on Perivascular Inflammation Index on Coronary CTA<br><br>Study Documents:	Title Acronym: COPIX<br><br>Other Ids: SDC 5327/21/102	Recruiting	• Atherosclerosis<br>• Inflammatory Response<br>• Coronary Disease	• Drug: Colchicine	Study Type: Interventional<br><br>Phase: Phase 1<br><br>Study Design:<br>• Allocation: Randomized<br>• Intervention Model: Parallel Assignment<br>• Masking: Single (Outcomes Assessor)<br>• Primary Purpose: Treatment<br><br>Outcome Measures:<br>• Quantification of FAI in both groups after 12-month follow-up<br>• Evaluation of the variation total atheroma volume<br>• Evaluation of low attenuation plate volume variation;<br>• Occurrence of general death<br>• Occurrence of cardiovascular death<br>• Occurrence of acute myocardial infarction<br>• Occurrence of stroke<br>• Occurrence of need for myocardial revascularization	Enrollment: 40<br><br>Age: 18 Years and older (Adult, Older Adult)<br><br>Sex: All	• University of Sao Paulo General Hospital	• Other	Study Start: March 7, 2022<br><br>Primary Completion: March 7, 2023<br><br>Study Completion: March 7, 2025<br><br>First Posted: April 26, 2022<br><br>Results First Posted: No Results Posted<br><br>Last Update Posted: May 9, 2022	• Heart Institute - University of São Paulo, São paulo, Sao Paulo, Brazil

	NCT Number	Title	Other Names	Status	Conditions	Interventions	Characteristics	Population	Sponsor/ Collaborators	Funder Type	Dates	Locations
2	NCT05250596	COLchicine On-admission to Reduce Inflammation in Acute Coronary Syndrome (COLOR-ACS)<br><br>Study Documents:	Title Acronym: COLOR-ACS<br><br>Other Ids:<br>• ID 20426<br>• 2021-000637-13	Recruiting	• Non ST Segment Elevation Acute Coronary Syndrome	• Drug: Colchicine<br>• Drug: Atorvastatin	Study Type: Interventional<br><br>Phase: Phase 2<br><br>Study Design:<br>• Allocation: Randomized<br>• Intervention Model: Parallel Assignment<br>• Masking: None (Open Label)<br>• Primary Purpose: Treatment<br><br>Outcome Measures:<br>• hsCRP change between admission and discharge<br>• Delta variation in creatinine value from baseline to peak<br>• Acute kidney injury incidence<br>• CK-MB peak value<br>• Glomerular filtration rate changes at 30 days after discharge<br>• Adverse clinical events from admission to 30 days after discharge<br>• Tolerance to colchicine	Enrollment: 175<br><br>Age: 18 Years and older (Adult, Older Adult)<br><br>Sex: All	• Azienda USL Toscana Centro	• Other	Study Start: February 24, 2022<br><br>Primary Completion: September 24, 2022<br><br>Study Completion: December 30, 2022<br><br>First Posted: February 22, 2022<br><br>Results First Posted: No Results Posted<br><br>Last Update Posted: March 16, 2022	• Gaia Chiara Selvaggia Magnaghi, Pescia, Italy<br>• Marco Comeglio, Pistoia, Italy<br>• Anna Toso, Prato, Italy

	NCT Number	Title	Other Names	Status	Conditions	Interventions	Characteristics	Population	Sponsor/ Collaborators	Funder Type	Dates	Locations
3	NCT05162742	Colchicine and Inflammation in Aortic Stenosis<br><br>Study Documents:	Title Acronym: CHIANTI<br><br>Other Ids: 112719	Recruiting	• Aortic Valve Disease<br>• Aortic Valve Stenosis<br>• Aortic Valve Calcification<br>• Inflammation<br>• Calcification<br>• Cardiovascular Diseases	• Drug: Colchicine<br>• Drug: Placebo	Study Type: Interventional<br><br>Phase: Phase 3<br><br>Study Design:<br>• Allocation: Randomized<br>• Intervention Model: Single Group Assignment<br>• Masking: Quadruple (Participant, Care Provider, Investigator, Outcomes Assessor)<br>• Primary Purpose: Treatment<br><br>Outcome Measures:<br>• Change in aortic valve calcium score<br>• Aortic valve 18F-NaF uptake<br>• Change in echocardiographic parameter for aortic stenosis<br>• Adverse Outcomes	Enrollment: 150<br><br>Age: 50 Years to 80 Years (Adult, Older Adult)<br><br>Sex: All	• Radboud University Medical Center	• Other	Study Start: November 1, 2022<br><br>Primary Completion: May 1, 2025<br><br>Study Completion: May 1, 2025<br><br>First Posted: December 17, 2021<br><br>Results First Posted: No Results Posted<br><br>Last Update Posted: October 26, 2022	• Radboudumc, Nijmegen, The Netherlands, Netherlands

	NCT Number	Title	Other Names	Status	Conditions	Interventions	Characteristics	Population	Sponsor/ Collaborators	Funder Type	Dates	Locations
4	NCT05130892	Effect of Inflammasome Inhibitor on hsCRP in Patients After PCI<br><br>Study Documents:	Title Acronym:<br><br>Other Ids:<br>NLRP3-CRP	Recruiting	•NLRP3<br>•hsCRP<br>•Percutaneous Coronary Intervention	•Drug: Colchicine<br>•Drug: Tranilast<br>•Drug: Oridonin	Study Type:<br>Interventional<br><br>Phase:<br>Phase 4<br><br>Study Design:<br>•Allocation: Randomized<br>•Intervention Model: Parallel Assignment<br>•Masking: None (Open Label)<br>•Primary Purpose: Treatment<br><br>Outcome Measures:<br>•Percentage change in hsCRP<br>•MACE (composite endpoint of all-cause death, nonfatal myocardial infarction, nonfatal stroke, revascularization due to ischemia, or hospitalization due to unstable angina pectoris)<br>•Bleeding<br>•Proteomics analysis	Enrollment:<br>132<br><br>Age:<br>18 Years to 80 Years (Adult, Older Adult)<br><br>Sex:<br>All	•Wuhan Union Hospital, China	•Other	Study Start:<br>November 15, 2021<br><br>Primary Completion:<br>August 31, 2022<br><br>Study Completion:<br>August 31, 2022<br><br>First Posted:<br>November 23, 2021<br><br>Results First Posted:<br>No Results Posted<br><br>Last Update Posted:<br>July 25, 2022	•Department of Cardiology, Union Hospital, Tongji Medical College, Huazhong University of Science and Technology, Wuhan, Hubei, China<br>•Wuhan Union Hospital, Wuhan, Hubei, China

	NCT Number	Title	Other Names	Status	Conditions	Interventions	Characteristics	Population	Sponsor/ Collaborators	Funder Type	Dates	Locations
5	NCT04857931	Colchicine in HFpEF<br><br>Study Documents:	Title Acronym: COLpEF<br><br>Other Ids: MP-33-2021-2929	Recruiting	•Heart Failure<br>•Inflammation<br>•Colchicine	•Drug: Colchicine<br>•Drug: Placebo	Study Type: Interventional<br><br>Phase: Phase 2<br><br>Study Design:<br>•Allocation: Randomized<br>•Intervention Model: Parallel Assignment<br>•Masking: Quadruple (Participant, Care Provider, Investigator, Outcomes Assessor)<br>•Primary Purpose: Treatment<br><br>Outcome Measures:<br>•Change in hs-CRP (C reactive protein)<br>•Change in circulating biomarkers of hemodynamic stress<br>•Change in circulating biomarkers of myocardial injury<br>•Change in left ventricular (LV) diastolic function<br>•Change in functional status and symptoms	Enrollment: 426<br><br>Age: 40 Years to 80 Years (Adult, Older Adult)<br><br>Sex: All	•Montreal Heart Institute	•Other	Study Start: June 17, 2022<br><br>Primary Completion: May 2024<br><br>Study Completion: October 2024<br><br>First Posted: April 23, 2021<br><br>Results First Posted: No Results Posted<br><br>Last Update Posted: July 7, 2022	•Montreal Heart Institute, Montréal, Quebec, Canada

	NCT Number	Title	Other Names	Status	Conditions	Interventions	Characteristics	Population	Sponsor/ Collaborators	Funder Type	Dates	Locations
6	NCT04848857	Colchine for the Stability of Coronary Plaque in Acute Coronary Syndrome(COLOCT)<br><br>Study Documents:	Title Acronym: COLOCT<br><br>Other Ids: CTACS202001	Recruiting	• Acute Coronary Syndrome	• Drug: Colchicine<br>• Drug: Placebo	Study Type: Interventional<br><br>Phase: Phase 4<br><br>Study Design:<br>• Allocation: Randomized<br>• Intervention Model: Parallel Assignment<br>• Masking: Double (Participant, Investigator)<br>• Primary Purpose: Treatment<br><br>Outcome Measures:<br>• changes of the Thickness of fibrous cap of coronary artery plaque<br>• changes of the Average lipid arc of coronary artery plaque<br>• changes of the Macrophage accumulation in coronary artery plaque<br>• changes of the Incidence of thin-cap fibroatheroma (TCFA)<br>• changes of the Minimum lumen area (MLA)<br>• changes of the percentage of lumen area stenosis<br>• changes of the inflammatory biomarker hsCRP<br>• changes of the cytokine IL-6<br>• the rate of All-cause death for acute coronary syndrome<br>• the rate of re-hospitalization for acute coronary syndrome	Enrollment: 128<br><br>Age: 18 Years to 80 Years (Adult, Older Adult)<br><br>Sex: All	• Wuhan Union Hospital, China	• Other	Study Start: May 3, 2021<br><br>Primary Completion: July 30, 2023<br><br>Study Completion: July 30, 2023<br><br>First Posted: April 19, 2021<br><br>Results First Posted: No Results Posted<br><br>Last Update Posted: July 25, 2022	• Wuhan Union Hospital, Wuhan, Hubei, China

	NCT Number	Title	Other Names	Status	Conditions	Interventions	Characteristics	Population	Sponsor/ Collaborators	Funder Type	Dates	Locations
7	NCT04616872	Treatment of Patients With Atherosclerotic Disease With Methotrexate-associated to LDL Like Nanoparticles<br><br>Study Documents:	Title Acronym:<br><br>Other Ids:<br>4786/19/005	Recruiting	• Atherosclerosis<br>• Coronary Artery Disease<br>• Inflammation	• Drug: Methotrexate-LDE<br>• Drug: Placebo-LDE	Study Type:<br>Interventional<br><br>Phase:<br>• Phase 2<br>• Phase 3<br><br>Study Design:<br>• Allocation: Randomized<br>• Intervention Model: Parallel Assignment<br>• Masking: Quadruple (Participant, Care Provider, Investigator, Outcomes Assessor)<br>• Primary Purpose: Treatment<br><br>Outcome Measures:<br>• Low Attenuation Plaque Volume (LAPV) coronary<br>• Low Attenuation Plaque Volume (LAPV) aortic<br>• Noncalcified plaque volume (NCPV)<br>• Dense calcified plaque volume (DCPV)<br>• Total lumen value (TLV)<br>• Remodeling index (RI)<br>• Perivascular fat attenuation index (FAI)<br>• Total atheroma volume (TAV)<br>• Total atheroma volume (TAV) aortic<br>• Clinical significant symptoms<br>• Other adverse events	Enrollment:<br>40<br><br>Age:<br>18 Years to 80 Years (Adult, Older Adult)<br><br>Sex:<br>All	• University of Sao Paulo General Hospital	• Other	Study Start:<br>October 10, 2020<br><br>Primary Completion:<br>October 12, 2022<br><br>Study Completion:<br>October 12, 2023<br><br>First Posted:<br>November 5, 2020<br><br>Results First Posted:<br>No Results Posted<br><br>Last Update Posted:<br>November 10, 2020	• Heart Institute (InCor) - University of São Paulo Medical School, São Paulo, Brazil, São Paulo, SP, Brazil<br>• Institute Prevent Senior, São Paulo, SP, Brazil

	NCT Number	Title	Other Names	Status	Conditions	Interventions	Characteristics	Population	Sponsor/ Collaborators	Funder Type	Dates	Locations
8	NCT04382443	Oral Colchicine in Argentina to Prevent Restenosis<br><br>Study Documents:	Title Acronym: ORCA<br><br>Other Ids: CECI-03	Recruiting	• Coronary Artery Disease<br>• Restenosis of Coronary Artery Stent<br>• Atherosclerosis<br>• Acute Coronary Syndrome	• Drug: Colchicine	Study Type: Interventional<br><br>Phase: Phase 4<br><br>Study Design:<br>• Allocation: Randomized<br>• Intervention Model: Parallel Assignment<br>• Masking: None (Open Label)<br>• Primary Purpose: Treatment<br><br>Outcome Measures:<br>• MACE<br>• Target lesion failure	Enrollment: 450<br><br>Age: 18 Years to 110 Years (Adult, Older Adult)<br><br>Sex: All	• Centro de estudios en Cardiologia Intervencionista	• Other	Study Start: March 12, 2020<br><br>Primary Completion: December 31, 2021<br><br>Study Completion: December 31, 2021<br><br>First Posted: May 11, 2020<br><br>Results First Posted: No Results Posted<br><br>Last Update Posted: March 18, 2021	• Sanatorio Otamendi, Ciudad de Buenos Aires, Buenos Aires, Argentina<br>• Sanatorio Las Lomas, San Isidro, Buenos Aires, Argentina

	NCT Number	Title	Other Names	Status	Conditions	Interventions	Characteristics	Population	Sponsor/ Collaborators	Funder Type	Dates	Locations
9	NCT04241601	Low-dose Interleukin-2 for the Reduction of Vascular Inflammation in Acute Coronary Syndromes - IVORY<br>Study Documents:	Title Acronym: IVORY<br>Other Ids:<br>• IVORY<br>• 2017-005130-27	Recruiting	• Acute Coronary Syndromes	• Drug: Interleukin-2 [IL-2]<br>• Other: Placebo Dextrose 5% solution	Study Type: Interventional<br>Phase: Phase 2<br>Study Design:<br>• Allocation: Randomized<br>• Intervention Model: Parallel Assignment<br>• Masking: Quadruple (Participant, Care Provider, Investigator, Outcomes Assessor)<br>• Primary Purpose: Treatment<br>Outcome Measures:<br>• Change in vascular inflammation<br>• Change in mean TBR max in each arterial region<br>• Change in lymphocyte subsets<br>• Change in percentage of Treg cells between low dose IL-2 and placebo<br>• Extended dosing of IL-2 in ACS patients safety and tolerability: Incidence of Adverse Events<br>• Extended dosing of IL-2 in ACS patients safety and tolerability: Incidence of injection site reaction<br>• Extended dosing of IL-2 in ACS patients safety and tolerability: Full Blood Count: total white cell count<br>• Extended dosing of IL-2 in ACS patients safety and tolerability: Full Blood Count: Red cell count<br>• Extended dosing of IL-2 in ACS patients safety and tolerability: Full Blood Count: Haemoglobin<br>• Extended dosing of IL-2 in ACS patients safety and tolerability: Full Blood Count: Platelets<br>• and 20 more	Enrollment: 60<br>Age: 18 Years to 85 Years (Adult, Older Adult)<br>Sex: All	• Cambridge University Hospitals NHS Foundation Trust	• Other	Study Start: August 5, 2020<br>Primary Completion: January 1, 2024<br>Study Completion: January 1, 2024<br>First Posted: January 27, 2020<br>Results First Posted: No Results Posted<br>Last Update Posted: June 2, 2022	• Addenbrooke's Hospital, Cambridge, Cambridgeshire, United Kingdom
